# Two Novel Norwithasteroids with Unusual Six- and Seven-Membered Ether Rings in Side Chain from Flos Daturae

**DOI:** 10.1155/2013/352019

**Published:** 2013-03-27

**Authors:** Bing-You Yang, Yong-Gang Xia, Yan-Yan Wang, Qiu-Hong Wang, Hai-Xue Kuang

**Affiliations:** Key Laboratory of Chinese Materia Medica of Ministry of Education, Heilongjiang University of Chinese Medicine, Harbin 150040, China

## Abstract

Chemical investigation of 50% ethanol eluate fraction of macroporous resin for the flower of *Datura metel* L. collected in Jiangsu province of China resulted in the isolation of two novel naturally occurring norwithasteroids, baimantuoluoline I (**1**) and baimantuoluoside J (**2**). Their structures were elucidated as 5**α**, 6**β**, 12**β**-trihydroxy-1-oxo-2-en-ergosta-21,24;22,29-diepoxy-26-carboxylic acid (**1**) and 5**α**, 6**β**, 12**β**, 25-tetrahydroxy-1-oxo-2-en-ergosta-21,24;22,29-diepoxy-26-carboxylic acid (**2**) on the basis of extensive spectroscopic analysis, including 1D, 2D-NMR, and HR-ESI-MS. According to the literatures, this study represents the first report of the norwithasteroids in the side chain with unusual six- and seven-membered ether rings instead of those with an unmodified skeleton (*δ*-lactone or *δ*-lactol side chain) and a modified skeleton (**γ**-lactone or **γ**-lactol side chain) in the family of withanolides. Meanwhile, compounds **1** and **2** were evaluated for their immunosuppressive activity against mice splenocyte proliferation in vitro.

## 1. Introduction

**Figure 1:**
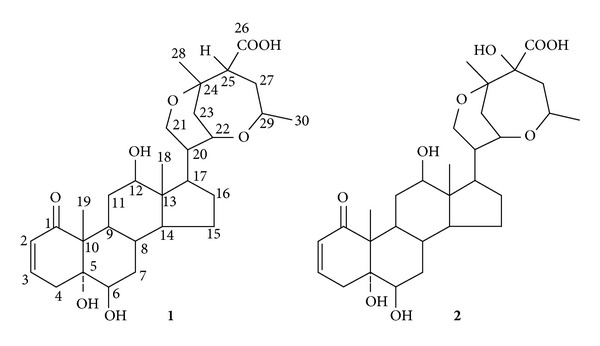
Structures of **1** and **2**.

Flos Daturae is the dry flower of *Datura metel* L. (Solanaceae), which widely distributed in Jiangsu, Guangdong, and Fujian province of China [1]. It has been used in traditional Chinese medicine for the treatment of asthma, convulsions, pain, and rheumatism for centuries [2]. It is said that it was almost one of the most important anesthetic of the ancient times. It has a very long history and is referred to in many early writings in China. For instance, according to the legend as early as 200 AD, a Chinese skilled doctor, Huatuo, once made use of “mafeisan” on patients in surgery [2, 3]. A recent study found that it has an obvious effect on the treatment of psoriasis [4, 5]. Moreover, it had been for clinical use in the first affiliated hospital of Heilongjiang University of Chinese Medicine, China [6, 7]. Our research group had done some work on its pharmacological actions for psoriasis and extraction and isolation of active constituents [5–8]. As a part of a continuing project to study the active constituents of Flos Daturae against psoriasis [3–5], we investigated 50% ethanol eluate fraction of macroporous resin for the flowers of *D. metel* L., which resulted in the discovery of two novel ergostane derivatives (Figure 1).

Naturally occurring withanolides are not widely distributed in the plant kingdom and isolated mainly from flowers, leaves, and seeds of Solanaceae plants [9–11]. Most of the withanolides may be subdivided into two subgroups: those with an unmodified skeleton (**δ**-lactone or **δ**-lactol side chain) and a modified skeleton (*γ*-lactone or *γ*-lactol side chain) [12, 13]. In this paper, we present the isolation and structural characterization of the two novel naturally occurring norwithasteroids on the basis of the interpretation of spectral data, including ^1^H-NMR, ^13^C-NMR, DEPT, ^1^H-^1^H-COSY, HSQC, HMBC, NOESY, and HR-ESI-MS. According to the literatures, this study represents the first report of naturally occurring norwithasteroids in the side chain with unusual six- and seven-membered ether rings formed rather than those with an unmodified skeleton (**δ**-lactone or **δ**-lactol side chain) and a modified skeleton (*γ*-lactone or *γ*-lactol side chain) in the family of withanolides. Meanwhile, compounds **1** and **2** were evaluated for their immunosuppressive activity against mice splenocyte proliferation in vitro.

## 2. Materials and Methods

### 2.1. General

IR spectra were recorded on a Shimadzu FTIR-8400S spectrometer. NMR spectra were recorded on a Bruker DPX 400 NMR instrument (400 MHz for ^1^H NMR and 100 MHz for ^13^C NMR). Chemical shifts are given as **δ** values with reference to tetramethylsilane (TMS) as internal standard, and coupling constants are given in Hz. HR-ESI-MS were carried out using IonSpec Ultima 7.0 T FTICR. Preparative HPLC (Waters, Delta 600–2487) was performed on a Hypersil-ODS II (10 m, 20 × 300 mm, Yilite, Dalian, China).

### 2.2. Plant Material

The dry flowers of *D. metel* were collected in Nanjing city of Jiangsu province of China in September 2002, and identified by prof. Zhenyue Wang. A voucher specimen (number 2002035) is deposited at the Herbarium of Heilongjiang University of Chinese Medicine, China.

### 2.3. Extraction and Isolation

The dried flowers (30 kg) of *D. metel* L. were extracted with 70% EtOH under reflux (2 × 100 L) for 2.5 h (each), and the combined solution was filtered and evaporated under vacuum to a syrup, followed by suspension in H_2_O. The suspension was acidified with 0.1% HCl and then filtered and exchanged for Styrene-DVB (001 × 7). The exchanged solution was passed through AB-8 Crosslinked Polystyrene and sequentially eluted with H_2_O, 50% EtOH, and 95% EtOH, respectively. 50% EtOH elution was concentrated under vacuum to yield a syrup (52.0 g), and this crude residue was subjected to silica gel and eluted successively with CHCl_3_/MeOH (10 : 1→1 : 1) gradient to give 10 fractions (Fr. 1–10). Fr. 7 (5 g) continues silica gel chromatography eluted with CHCl_3_/MeOH (5 : 1→1 : 1) to afford a number of subfractions A_1_–A_13_. Compounds **1** (45 mg, *t*_R_ = 26.9 min) and **2** (39 mg, *t*_R_ = 21.7 min) were obtained by prep. HPLC chromatography of the sub-fraction A_7_ (0.5 g) with MeOH/H_2_O (2 : 3). 

Baimantuoluoline I (**1**): white amorphous powder, [*α*]^25^_D_ = +12.3 (c = 0.1, MeOH). IR (KBr): *ν*_max⁡_ = 3423, 2921, 1698, 1384, 1281, 1132, 1091, 992 cm^−1^. HR-ESI-MS (positive): *m/z* = 533.30958 (cal. for C_30_H_45_O_8_ 533.31144, [M+H]^+^), 555.29130 (cal. for C_30_H_44_NaO_8_, 555.29339, [M+Na]^+^) and 571.26598 (cal. for C_30_H_44_KO_8_, 571.26733, [M+K]^+^). ^1^H and ^13^C-NMR: see Table 1.

Baimantuoluoside J (**2**): white amorphous powder, [*α*]^25^_D_ = +18 (c = 0.1, MeOH). IR (KBr): *ν*_max⁡_ = 3407, 3323, 2940, 2917, 2855, 2578, 1675, 1068, 1033 cm^−1^. HR-ESI-MS (positive): *m/z* = 549.30349 (cal. for C_30_H_45_O_9_ 549.30636, M+H]^+^), 571.28576 (cal. for C_30_H_44_NaO_9_, 571.28830, [M+Na]^+^) and 587.25940 (cal. for C_30_H_44_KO_9_, 587.26224, [M+K]^+^). ^1^H and ^13^C-NMR: see Table 1.

### 2.4. Con A-Induced Mouse Splenocyte Proliferation

Mouse splenocyte proliferation was assayed by MTT method as previously described [14]. Splenocytes were seeded into a 96-well flat-bottom microtiter plate at 1 × 10^8^ cells/mL in 100 *μ*L of complete medium. Serial drug dilutions were prepared in medium immediately prior to each assay. Thereafter, 100 *μ*L aliquots of serial dilution of each test compound were added (parallel triplicate wells were set), and then the cells were incubated in the absence or presence of Concanavalin A (Con A, final concn. 5 *μ*g/mL) for 44 h in the humidified 5% CO_2_ incubator at 37°C. MTT (3-(4,5-dimethylthiazol-2-yl)-2,5-diphenyl tetrazolium bromide) in phosphate buffered saline (PBS) at 5 mg/mL (10 *μ*L) was added to each well, plates were incubated at 37°C for 4 h, and the formazan crystals formed were dissolved through addition of 100 *μ*L of DMSO/well. The absorption of the samples was measured using an ELISA reader (Uniscan Titertec) at a wavelength of 570 nm. Cyclosporine was used for positive control. The immunosuppressive activity of each compound was expressed as the concentration that inhibited splenocyte proliferation to 50% (IC_50_) of the control value.

## 3. Results and Discussion

### 3.1. Structural Elucidation of Compound **1**

Compound **1** was obtained as a white amorphous powder and showed negative results for the Molish reagent and positive results for the Liebermann-Burchard reaction, which indicated that there may be a triterpenoid or steroid aglycone. Its molecular formula was established as C_30_H_44_O_8_ by the positive HR-ESI-MS from [M+H]^+^ at *m/z* 533.30958 (cal.533.31144), [M+Na]^+^ at *m/z* 555.29130 (cal. 555.29339), and [M+K]^+^ at *m/z* 571.26598 (cal. 571.26733), indicating 9 degrees of unsaturation.

The ^1^H-NMR spectrum of **1** (Table 1) showed several characteristic signals in A-D rings of steroid skeleton. Two signals at **δ** 0.77 (3H, s) and 1.30 (3H, s) were attributed to Me-18 and Me-19, respectively. A Me-21 signal was missing and was replaced by two doublets at **δ** 3.47 (1H, br. t, J = 12.0 Hz) and 3.86 (1H, d, J = 12.0, 5.2 Hz), suggesting that C-21 was substituted by the oxygen-bearing group. Additional two methyl signals at **δ**1.33 (3H, s) and 1.37 (3H, d, J = 6.0 Hz) were undisputed to be assigned to the side chain. The vinylic hydrogen signals at **δ** 5.77 (1H, dd, J = 10.1, 2.4 Hz) and *δ* 6.65 (1H, ddd, J = 10.1, 5.1, 2.1 Hz) were attributed to H-2 and H-3 protons, respectively, in a steroidal Δ^2^-1-one system. The multiplicity of the H-2 and H-3 signals indicated that position C-4 was unsubstituted. A triplet resonating at *δ* 3.51 (1H, t, J = 2.2 Hz) was due to the oxygenated C-6 methine proton. The **β**-configuration of the hydroxyl at C-6 was established by a NOESY experiment. The NOESY spectrum showed the correlation between Me-19 and H-4**β** and between H-6 and H-4**α**, indicating that H-6 has an **α**-configuration. Besides, there is a double doublet at **δ** 3.44 (1H, dd, J = 10.8, 4.4 Hz) in ^1^H-NMR spectrum. Its coupling constants and splitting pattern were characteristic of 12**β**-hydroxywithanolide [3–5].

**Figure 2:**
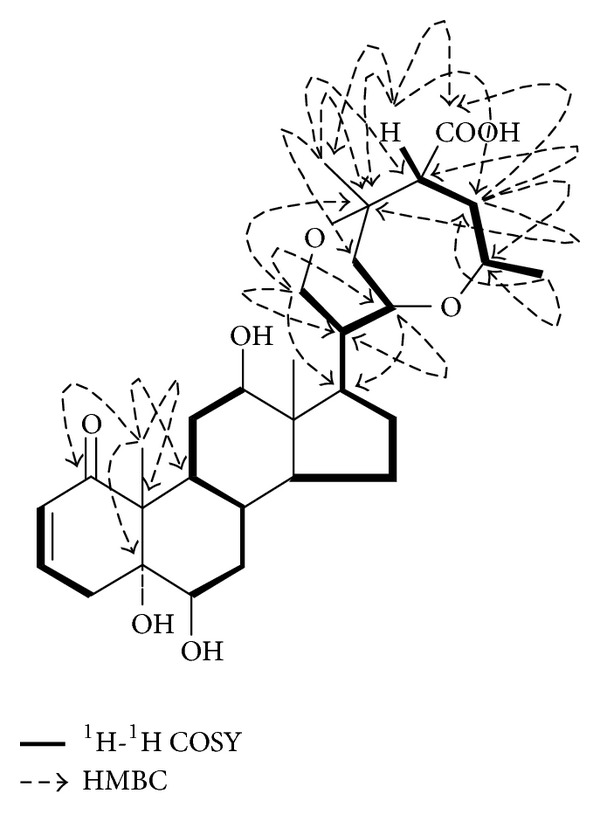
Key ^1^H-^1^H COSY and HMBC correlations of **1**.

The ^13^C-NMR spectrum of **1** showed resonances for all 30 carbons (Table 1). The carbon signals from C-1 to C-21 were easily assigned according to 2D-NMR and the comparison of similar substituted patterns of withanolides from the A ring to the D ring. The characteristic downfield signals at *δ* 207.3 were due to ketone carbonyl, along with the characteristic doublets at *δ* 128.9 and 144.0 for the vinylic carbons at C-2 and C-3, respectively, in the ring A. The signals appearing at **δ**16.2 and 9.2 were assigned to the Me-19 and Me-18, respectively. The typical signals at *δ*78.3, 75.2, 79.8.0, and 65.5 were assigned to the oxygenated carbons at C-5, C-6, C-12, and C-21, respectively. The chemical shifts of the C-5 and C-6 further indicate the presence of a 5**α**, 6**β**-dihydroxyl steroid residue because their chemical shifts and multiplicity agreed with a similar substitution pattern [4]. The downfield chemical shifts of C-21 suggested that there may be such a 21, 24 epoxy structure segment in the side chain. In that case, a tetrahydropyrane ring was proposed, including C-20 (*δ* 44.9), C-21 (*δ*65.5), C-22 (*δ* 68.3), C-23 (*δ* 40.8), and C-24 (*δ* 75.5). This tetrahydropyrane ring plays an important role in connection of steroid mother nucleus and the side chain. Further support this assumption was obtained that a serial of long-range correlations between H-21 [*δ* 3.47 (1H, br. t, J = 11.6 Hz) and 3.86 (1H, dd, J = 11.6, 5.2 Hz)] and C-17, C-20, C-22, and C-24, between H-22 [*δ* 3.89 (1H, dd, J = 10.4, 4.8 Hz)] and C-17, C-20, and C-24, and between H-23 [*δ*1.93 (1H, br. t, J = 12.8 Hz) and 1.71 (1H, dd, J = 12.8, 4.4 Hz)] and C-20, C-22, and C-24 (Figure 2).

Except for confirmed 24 carbon signals mentioned above, additional six carbon signals should be elucidated in the side chain, including *δ*178.2 (C), 75.8 (CH), 52.3 (CH), 33.6 (CH_2_), and 21.1 (CH_3_) assigned by DEPT spectrum. The characteristic downfield signals at *δ* 178.2 and 75.8 were due to free carboxyl group and oxygenated carbon signals, respectively. The signal appearing at **δ**21.1 was assigned to the two methyl carbon signals [*δ*H, 1.33 (3H, s) and 1.37 (3H, d, J = 6.0 Hz)] according to its HSQC spectrum. By ^1^H-^1^H COSY spectrum, a structural segment was confirmed as the –CH [*δ* 2.92(1H, dd, J = 12.0, 8.8 Hz)]–CH_2_ [*δ* 2.41 and 1.83 (each, 1H, m)]–CH [*δ* 4.46 (1H, dt, J = 10.0, 5.8 Hz)]–O– residue (Figure 2). Taking into account the NMR spectral data and the 9 degrees of unsaturation calculated from the empirical formula of compound **1**, it was suggested that it is possible to be presence of another ether ring except for an **α**, **β**-unsaturation carbonyl group, four rings of steroid skeleton, one tetrahydropyrane ring, and one carboxyl. In that case, another seven-membered-ether ring was proposed as shown in Figure 1. Further support this assumption was obtained that a serial of long-range correlations between H-25 [*δ*2.92 (1H, dd, J = 12.0, 8.8 Hz)] and C-24, C-26, C-27, and C-29, between H-27 [*δ*2.41 and 1.83 (each, 1H, m)] and C-24, C-25, C-26, and C-29, between Me-28 [*δ*1.33 (3H, s)] and C-23, C-24, and C-25, and between Me-30 [*δ*1.37 (3H, d, J = 6.0 Hz)] and C-29 and C-27. Thus, an oxepane ring moiety was determined in ring E, as shown in Figure 2. Therefore, the structure of **1** was deduced as 5**α**, 6**β**, 12**β**-trihydroxy-1-oxo-2-en-ergosta-21,24;22,29-diepoxy-26-carboxylic acid, which was named baimantuoluoline I.

### 3.2. Structural Elucidation of Compound **2**

Compound **2** was obtained as a white amorphous powder and showed negative results for the Molish reagent and positive results for the Liebermann-Burchard reaction. Its molecular formula was established as C_30_H_44_O_9_ by the positive HR-ESI-MS from [M+H]^+^ at *m/z* 549.30349 (cal. 549.30636), [M+Na]^+^ at *m/z* 571.28576 (cal. 571.28830), and [M+K]^+^ at *m/z* 587.25940 (cal. 587.26224), indicating 9 degrees of unsaturation.

The ^1^H-NMR spectrum of **2** (Table 1) showed distinct resemblance to those of baimantuoluoside I. The only notable difference was the change of H-25 signal, which was missing. The ^13^C-NMR (DEPT) spectrum showed an additional downfield C-atom signal at **δ** 82.2 in **2** (Table 1), which was affirmatively assigned to the C-25, indicating that C-25 was substituted by a hydroxyl group. On the basis of previous data, the structure of **2** was identified to be 5**α**, 6**β**, 12**β**, 25-tetrahydroxy-1-oxo-2-en- ergosta-21,24;22,29-diepoxy-26-carboxylic acid, which was named baimantuoluoline J.

### 3.3. Possible Biosynthetic Pathway to Proposed Structures

**Figure 3:**
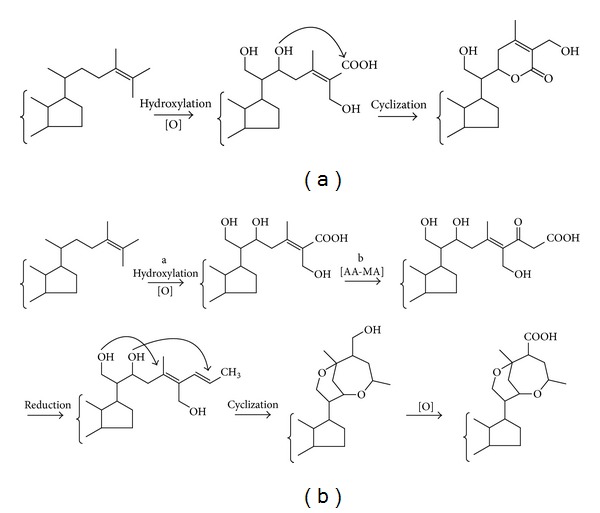
Possible biosynthetic pathway to common withanolides (a) and proposed structures (b). The order of a and b may be interchangeable.

The biosynthetic pathway of common withanolides in plants is derived from phytosterol, which possibly experienced a sequential oxidation, hydroxylation, and cyclization in the side chains and finally formed the basic skeleton of withanolides (Figure 3(a)) [12]. For compounds **1** and **2**, they have two more carbon atoms than the common withanolide compounds in the side chain, which is never to be seen in other phytosterol compounds. A logical biosynthetic pathway of compounds **1** and **2** is postulated as shown in Figure 3(b). The compounds **1** and **2** may be derived from the ordinary phytosterol compounds as precursors that firstly experience hydroxylation and oxidation and along with an acetate-malonate (AA-MA) pathway resulting in the side chains increase two carbon atoms and finally involve a complex process such as reduction, cyclization, and oxidation to form this kind of unusual six- and seven-membered ether rings in side chain. This type of biosynthetic pathway is rarely seen, and detailed evidences also need to be further confirmed.

### 3.4. Effect of Compounds on the Splenocyte Proliferation of Mouse

The colorimetric assay using MTT for cell proliferation was then carried out to evaluate Con A-stimulated mouse splenocyte in the presence of various concentrations of **1** and **2**. The results of this assay showed that compounds **1** and **2** possess the obvious immunosuppressive activity with the IC_50_ values of 10.2 nM and 13.5 nM (Table 2), respectively. However, their activities were much lower than that of the positive medicine cyclosporine. Compounds **1** and **2** did not show cytotoxicity in the range of tested concentrations, indicating that the immunosuppressive effects observed in vitro may be not due to the toxicity of compounds.

## 4. Conclusion

Over the period of August 1996 to March 2010, 360 new naturally occurring withanolides were isolated largely from the flowers, leaves, and seeds of Solanaceae plants such as *Withania somnifera*, *W. cagalans, W. aristata, Physalis pubescens, P. peruviana, P. minima, D. ferox, D. fastuosa, D. inoxia, D. metel, and D. stramoniam*, [9–11]. The characteristic feature of their skeleton of withanolides can be summarized as the two subgroups: those with an unmodified skeleton (**δ**-lactone or **δ**-lactol side chain) and a modified skeleton (**γ**-lactone or **γ**-lactol side chain) [12], which have been shown to be associated with many biological activities including cytotoxic, anti-inflammatory, antioxidant, antiarthritic, anticholinesterase, immunoprotective, trypanocidal, antimalarial, leishmanicidal, and diuretic [13].

However, till now, no studies on ergostane derivatives with unusual six- and seven-membered ether rings formed in the side chain have been found in the family of Solanaceae. At the same time, these two naturally occurring norwithasteroids have been found to possess immunosuppressive activity by mice splenocyte proliferation in vitro. It is worthy of mentioning that tropane alkaloids have for a long time been considered as characteristic ingredients of Flos Daturae [12]. This finding revealed another kind of biological constituents of unusual norwithasteroids in Flos Daturae, enlarged our understanding of Flos Daturae against psoriasis, and was possible to uncover the real material foundation of Flos Daturae against psoriasis.

## Figures and Tables

**Table 1:** ^1^H and ^13^C-NMR data of **1** and **2** in CD_3_OD at 400 MHz and 100 MHz, *J *in Hz.

Number	**1**		**2**	
	*δ*_C_	*δ*_H_	*δ*_C_	*δ*_H_
1	207.3		207.3	
2	128.9	5.77 dd (10.0, 2.4)	128.9	5.77 dd (10.1, 2.4)
3	144.0	6.65 ddd (10.0, 5.2, 2.1)	144.0	6.65 ddd (10.1, 5.1, 2.1)
4	36.5	3.24 dt (20.0, 2.4)	36.5	3.24 dt (20.0, 2.4)
		2.03 dd (20.0, 5.2)		2.03 dd (20.0, 5.1)
5	78.3		78.3	
6	75.2	3.51 t (2.4)	75.2	3.51 t (2.2)
7	33.7	1.55 (m)	33.7	1.55 (m)
		1.63 (m)		1.64 (m)
8	30.5	1.67 (m)	30.4	1.68 (m)
9	41.2	1.86 (m)	41.3	1.85 (m)
10	52.9		52.9	
11	34.3	2.39 dt (12.6, 3.8)	34.2	2.37 dt (12.4, 3.8)
		1.32 (m)		1.32 (m)
12	79.8	3.44 dd (10.8, 4.4)	79.9	3.42 dd (11.6, 3.6)
13	48.4		48.4	
14	55.3	1.10 (m)	55.3	1.09 (m)
15	24.7	1.25 (m)	24.7	1.25 (m)
		1.68 (m)		1.68 (m)
16	25.5	1.68 (m)	25.5	1.68 (m)
		2.04 (m)		2.08 (m)
17	52.2	1.48 (m)	52.3	1.44 (m)
18	9.2	0.77 (3H, s)	9.2	0.76 (3H, s)
19	16.2	1.30 (3H, s)	16.2	1.29 (3H, s)
20	44.9	1.80 (m)	45.0	1.78 (m)
21	65.5	3.47 br. t (11.6)	66.1	3.40 br. t (12.0)
		3.86 dd (11.6, 5.2)		3.85 dd (12.0, 5.2)
22	68.3	3.89 dd (10.4, 4.8)	68.7	3.82 dd (11.2, 4.8)
23	40.8	1.71 dd (12.8, 4.4)	39.3	1.89 dd (12.8, 4.8)
		1.93 br. t (12.8)		1.99 br. t (12.8)
24	75.5		80.1	
25	52.3	2.92 dd (12.0, 8.8)	82.2	
26	178.2		179.9	
27	33.6	2.41 (m)	42.3	2.86 dd (13.6, 6.4)
		1.83 (m)		1.72 dd (13.6, 9.2)
28	21.1	1.33 (3H, s)	17.0	1.31 (3H, s)
29	75.8	4.46 dt (10.0, 5.8)	75.2	4.54 dt (9.2, 6.2)
30	21.1	1.37 d (3H, 6.0)	22.0	1.33 d (3H, 6.0)

**Table 2:** Effect of compounds **1** and **2** on Con A proliferation of mouse splenocytes in vitro.

Compounds	IC_50_ (nM)
Cyclosporine	4.3 ± 0.2
**1**	10.2 ± 1.3
**2**	13.5 ± 1.6

## Abbreviations

DEPT: Distortionless enhancement by polarization transfer;
COSY: Correlation spectroscopy
HSQC: ^1^H detected heteronuclear single quantum coherence
HMBC: ^1^H detected heteronuclear multiple bond correlation
NOESY: Nuclear overhauser effect spectroscopy
AA-MA: Acetate-malonate pathway.

## References

[1] State Pharmacopoeia Committee (2010). *Pharmacopoeia of People’s Republic of China*.

[2] Jiangsu New Medical college (1999). *Zhong Yao Da Ci Dian*.

[3] Kuang H, Yang B, Tang L, Xia Y, Dou D (2009). Baimantuoluosides A-C, three new withanolide glucosides from the flower of *Datura metel* L. *Helvetica Chimica Acta*.

[4] Yang B, Wang Q, Xia Y, Feng W, Kuang H (2008). Baimantuoluolines D-F, three new withanolides from the flower of *Datura metel* L. *Helvetica Chimica Acta*.

[5] Yang BY, Xia YG, Wang QH, Dou DQ, Kuang HX (2010). Baimantuoluosides D-G, four new withanolide glucosides from the flower of *Datura metel* L. *Archives of Pharmacal Research*.

[6] Wang QH, Xiao HB, Yang BY, Yao FY, Kuang HX (2008). Studies on pharmacological actions of the effective parts for psoriasis in Flos Daturae (I). *China Journal Experimental Traditional Medical Formulae*.

[7] Wang YX (1985). the report on Traditional Chinese medicine yangjinhua (*Datura metel*) is given priority to treat 242 patients with psoriasis. *Journal of Traditional Chinese Medicine*.

[8] Kuang HX, Yang BY, Xia YG, Wang QH (2011). Two new withanolide lactones from flos daturae. *Molecules*.

[9] Glotter E, Kirson I, Abraham A, Lavie D (1973). Constituents of *Withania somnifera* Dun—13. The withanolides of chemotype III. *Tetrahedron*.

[10] Pan Y, Wang X, Hu X (2007). Cytotoxic withanolides from the flowers of *Datura metel*. *Journal of Natural Products*.

[11] Benjumea D, Martín-Herrera D, Abdala S (2009). Withanolides from *Whitania aristata* and their diuretic activity. *Journal of Ethnopharmacology*.

[12] Chen LX, He H, Qiu F (2011). Natural withanolides: an overview. *Natural Product Reports*.

[13] Mirjalili MH, Moyano E, Bonfill M, Cusido RM, Palazón J (2009). Steroidal lactones from *Withania somnifera*, an ancient plant for novel medicine. *Molecules*.

[14] Kuang H, Xia Y, Yang B, Wang Q, Wang Y (2011). Screening and comparison of the immunosuppressive activities of polysaccharides from the stems of *Ephedra sinica* Stapf. *Carbohydrate Polymers*.

